# 

**Published:** 1872-04-01

**Authors:** 


Money Receipts up to April 1st, 1872.—H. Sweet, $6; A. Groesbeck, $3; N. Bridge, $3; J.W. Barlow, $3; E. R. Bacon, $3; C. H. Bacon, $6; W. Martin, $3; O. N. Moon, $3; W. Dougal, $6; J. H. Rauch, $6; J. P. Johnson, $3; J. G. Conley, $3; J. W. Boggess, $3; R. J. Patterson, $3; M. Northup, $3; A. Jones, $3; L. D. Thompkins, $3; Dr. Sprague, $3; J. L. Shay, $3; W. J. Johnson, $3; Wm. Butterfield, $3; H. John, $3.

